# A banana susceptibility gene underlying fusarium wilt: validation as a target for disease resistance

**DOI:** 10.1007/s11033-026-12689-8

**Published:** 2026-08-29

**Authors:** Márcio Leandro da Silveira Fonseca, Luiz Carlos de Souza Júnior, Fernanda dos SantosNascimento, Andresa Priscila de Souza Ramos, Welly Sacramento Santana, Marcelly Santana Mascarenhas, Edson Perito Amorim, Claudia Fortes Ferreira

**Affiliations:** 1 Universidade Federal do Recôncavo da Bahia, Rua Rui Barbosa, 710, Centro, Cruz das Almas, 44380-000 Bahia, Brazil; 2 Embrapa Mandioca e Fruticultura, Rua Embrapa s/n, C.P. 007, Centro, BA 44380-000 Cruz das Almas, Brazil; 3 Universidade Estadual de Feira de Santana, Avenida Transnordestina, s/n, Bairro Novo Horizonte, Feira de Santana, 44036-900 BA Brazil

**Keywords:** *Musa* spp., Genetic improvement, CRISPR/Cas, DMR6, Molecular markers

## Abstract

**Background:**

*Fusarium* wilt, caused by the soil-borne fungus *Fusarium oxysporum* f. sp. *cubense*, remains the principal constraint on banana production, particularly for the widely cultivated cultivar ‘Prata-Ana’ (AAB) in Brazil. Given the limited efficacy of conventional control strategies, susceptibility (S) genes have emerged as promising targets for developing resistant cultivars. This study investigated the expression of the banana DMR6 gene during the interaction with Foc. DMR6 was selected because it is a conserved plant susceptibility gene that negatively regulates salicylic acid-mediated immunity, making it a promising target for genome editing.

**Methods and Results:**

Banana plantlets were inoculated with Foc Subtropical Race 4 under controlled conditions. Temporal expression of the banana DMR6 gene was analyzed by RT-qPCR, and host defense responses were assessed by histochemical and microscopic analyses. DMR6 expression initially decreased and subsequently increased, reaching a 6.5-fold induction at 72 h post-inoculation relative to non-inoculated controls. This expression peak coincided with spore formation and advanced vascular colonization. Although infected roots exhibited callose deposition and phenolic compound accumulation, these defense responses were insufficient to restrict pathogen progression, resulting in severe disease symptoms and a disease severity index of 80% at 90 days after inoculation.

**Conclusions:**

The findings indicate that banana DMR6 functions as a negative regulator of plant immunity and is closely associated with susceptibility to Fusarium wilt. These results provide a molecular basis for future functional validation and support DMR6 as a potential target for precise genome editing to develop resistant banana cultivars.

## Introduction

Bananas (*Musa* spp.) are among the most important food crops world wide with production exceeding 139 million tons in 2024 across approximately 6.0 million hectares [1]. Its wide adaptability enables cultivation throughout tropical and subtropical regions; however, productivity is severely constrained by phytosanitary challenges. Major global threats include yellow and black Sigatoka, bacterial wilt, and, most critically, Fusarium wilt [2–4].

Fusarium wilt, caused by the soil borne fungus *Fusarium oxysporum* f. sp. *cubense* (Foc), is the most devastating disease affecting global banana production [5]. As a hemibiotrophic pathogen, Foc initially colonizes living tissues before transitioning to an aggressive necrotrophic phase, leading to vascular blockage, leaf chlorosis, and eventual plant death [3]. Its long-term persistence in soil through chlamydospores renders infested areas unsuitable for cultivation for decades, underscoring both its severity and epidemiological impact [6].

The development of resistant cultivars represents the most effective and sustainable strategy for managing Fusarium wilt, minimizing yield losses and environmental impacts [3]. In Brazil, the Banana Genetic Breeding Program at Embrapa Mandioca e Fruticultura plays a pivotal role, particularly through the development of hybrids within the Prata subgroup by crossing improved diploids with commercial cultivars to introduce resistance genes [7].

However, conventional banana breeding is hindered by polyploidy and sterility, which limit recombination and prolong breeding cycles, often compromising fruit quality. Given the rapid evolution of pathogens, integrating biotechnological approaches is essential to accelerate the development of resistant cultivars while preserving key agronomic and organoleptic traits [8, 9].

Among biotechnological tools, CRISPR/Cas9 has emerged as a powerful strategy for precise genome editing [10–12]. In bananas, this approach is particularly promising because it enables the direct improvement of elite polyploid cultivars by disrupting specific genes while preserving their genetic background [13, 14]. However, the successful application of this technology depends on the identification and functional characterization of suitable target genes, particularly susceptibility (S) genes whose expression is modulated during pathogen infection. The identification of susceptibility genes (S-genes) is critical for developing targeted resistance strategies. In this context, transcriptional profiling during pathogen infection has emerged as a important approach for identifying candidate S-genes whose differential expression may indicate their involvement in host susceptibility [15]. In bananas, although CRISPR/Cas9 has been successfully applied against viral and bacterial pathogens [13, 16, 17], the expression dynamics of S-genes during infection by fungal pathogens such as Foc remain poorly understood.

Because the timing and magnitude of gene expression can provide important insights into the role of candidate susceptibility genes during plant–pathogen interactions, characterizing the expression profile of MusaDMR6 during Foc infection represents an important step toward evaluating its potential as a target for genome-editing strategies. The integration of gene editing with genomic screening and molecular marker approaches is critical for identifying effective targets in bananas [18, 19]. Among these, the susceptibility gene DMR6 (Downy Mildew Resistance 6), which encodes a 2-oxoglutarate Fe (II)-dependent oxygenase, has emerged as a key candidate because it negatively regulates salicylic acid-mediated defense responses [20]. In several plant species, loss-of-function mutations in DMR6 enhance resistance to bacterial, fungal, and oomycete pathogens, while in *Musa* spp. the induction of the ortholog *MusaDMR6* during pathogen infection suggests a similar role in susceptibility. Therefore, characterizing its expression is a crucial step toward validating knockout-based resistance strategies [20, 21]. The manipulation of S-genes has progressed from model species to major crops focusing on genes that regulate basal immunity [22, 23]. Notably, CRISPR/Cas9 mediated knockout of DMR6 has conferred effective resistance to hemibiotrophic pathogens in crops such as potato and tomato, without significant penalties to plant growth or development [24, 25].

Previous studies have demonstrated that MusaDMR6 knockout lines generated via CRISPR/Cas9 confer enhanced resistance to *Xanthomonas campestris* pv. musacearum, providing functional evidence that MusaDMR6 acts as a susceptibility gene in banana [21, 26]. However, these findings are limited to bacterial pathogens, and whether MusaDMR6 exhibits a similar transcriptional response during infection by *Fusarium oxysporum* f. sp. *cubense* remains unknown, particularly in the commercially important ‘Prata-Ana’ (AAB) cultivar. Therefore, this study aimed to characterize the transcriptional profile of MusaDMR6 during the interaction between ‘Prata-Ana’ and Foc. Using RT-qPCR, gene expression was evaluated at different stages of root infection to determine whether the transcriptional regulation of MusaDMR6 is associated with pathogen colonization and disease progression. These findings provide a molecular basis for future functional validation of MusaDMR6 and support its evaluation as a potential target for precision breeding strategies based on CRISPR/Cas9 genome editing.

## Materials and methods

### Plant material and experimental design

The experiment was conducted at the experimental station of Embrapa Mandioca e Fruticultura (Cruz das Almas, Bahia, Brazil). A total of 35 banana (*Musa* spp.) plantlets of the ‘Prata-Ana’ (AAB) cultivar were used. Plants were previously acclimatized and transplanted into plastic pots containing a substrate composed of pine bark (Tecnomax^®^) and coconut fiber (5:1, v: v), and maintained for 30 days under cultivation conditions. The experimental design was completely randomized with three biological replicates per treatment. The remaining plants were used for disease severity evaluation at 90 days after inoculation.

### Selection of the targeted gene

The DMR6 gene was selected based on consistent genetic, transcriptional, and functional evidence supporting its conserved role as a negative regulator of plant immunity and its suitability as a target for genome editing [21, 27]. In bananas, Musa DMR6 has been associated with susceptibility to biotic stress, reinforcing its relevance for functional characterization in plant pathogen interactions.

### Preparation of Foc inoculum and substrate infestation

The pathogen used in this study was Foc isolate CNPMF 218 (Subtropical Race 4, STR4), obtained from the Biological Collection of Microorganisms of the Plant Pathology Laboratory at Embrapa Mandioca e Fruticultura. This isolate was selected because of its high virulence toward banana plants [28]. The fungus was reactivated on potato dextrose agar (PDA; 200 g L⁻¹ potato, 20 g L⁻¹ dextrose, and 20 g L⁻¹ agar) and incubated at 25 °C under a 12 h photoperiod for 7 days. Conidia were harvested by gently scraping the colony’s surface with sterile brushes and suspending them in sterile deionized water.

For inoculum production, 10 mL of the conidial suspension was transferred to 500 g of previously autoclaved rice (120 °C for 20 min) and incubated for 15 days at 25 °C under a 12 h photoperiod to allow fungal colonization. The concentration of the rice-based inoculum was determined by colony-forming unit (CFU) counts after serial dilution and adjusted to 1 × 10⁶ CFU g⁻¹ of colonized rice. Finally, approximately 40 g of the colonized rice inoculum was thoroughly mixed with the substrate in each pot to ensure uniform infestation before transplanting the banana plantlets.

### Sampling procedure

Plant material was collected at 0, 24, 48, and 72 h post-inoculation (hpi). At each time point, three inoculated and three non-inoculated plants (controls) were sampled. Root and leaf fragments (~ 1 cm) were collected per plant. At 90 days after inoculation (DAI), disease severity was visually assessed by longitudinally sectioning the rhizomes and scoring the extent of internal vascular discoloration on a 1–5 rating scale, according to [29]. The Internal Damage Index (IDI- $\:DI\left(\%\right)=$ $\left[\frac{\sum\:\left(\mathrm{disease\:rating}\times\:\mathrm{number\:of\:plants\:with\:this\:rating}\right)\times\:100}{\mathrm{total\:number\:of\:plants\:evaluated\:per\:plot}\times\:\mathrm{maximum\:rating\:in\:the\:scale}}\right]$) was subsequently calculated according to the method described by McKinney (1923) [30]. Samples for histological analysis were fixed in Karnovsky’s solution [31] and stored at 4 °C. Samples for gene expression analysis were immediately frozen in liquid nitrogen and stored at − 80 °C until RNA extraction.

### Histochemical analyses

Root samples were fixed in Karnovsky’s solution for 48 h, dehydrated in an ethanol series (50–100%) and embedded in hydroxyethyl methacrylate resin (Historesin^®^, Leica). Polymerization was performed at room temperature for 72 h. Section  (8 μm) were obtained using a rotary microtome (Leica RM1516) and mounted on glass slides. Microscopic observations and image acquisition were performed using an Olympus BX51 microscope.

Fungal colonization was assessed following adapted protocols [32]. Root fragments were cleared in 10% KOH (48 h), acidified in 1% HCl (5 min), and stained with 0.05% Trypan blue in lactoglycerol (2:1:1, v: v:v) for 1 h. Samples were subsequently stored in lactoglycerol and analyzed under bright field microscopy. Phenolic compounds were detected using ferric chloride staining according to [33].

### RNA extraction and cDNA synthesis

Total RNA was extracted from root tissues using a modified CTAB protocol [34]. RNA quality and integrity were assessed by electrophoresis on 1% agarose gels and spectrophotometric analysis using a NanoDrop™ ND-ONE (Thermo Scientific). Genomic DNA contamination was removed using TURBO DNase (Ambion^®^) following the manufacturer’s instructions. RNA integrity after treatment was confirmed by agarose gel electrophoresis. First-strand cDNA was synthesized from 5 µL of total RNA using the high capacity RNA-to-cDNA™ kit in a 20 µL reaction volume, following the thermal profile: 37 °C for 60 min, 95 °C for 5 min, and hold at 4 °C. cDNA samples were stored at − 20 °C.

### RT-qPCR analysis

Gene expression analysis was performed using RT-qPCR with gene-specific primers related to plant–pathogen interactions (Table 1). Primers targeting MusaDMR6 were included based on previous validation of its role in immune modulation in banana [21].

**Table 1:** Primers used for gene expression evaluation in the *Musa* spp. x *Fusarium oxysporum* f. sp. *cubense* interaction

ID	Sequence (5′-3′)	Amplicon	Reference
MusaDMR6	F: TTATTGATGAAATGGTGAGCGTGG	164 bp	Designed based on the MusaDMR6 gene (NCBI genbank).
	R: ATGCAGGCGCAGATAATCGCGCCA		
25s	F: ACATTGTCAGGTGGGGAGTT	106	[35]
	R: CCTTTTGTTCCACACGAGATT		
*RPS2*	F: TAGGGATTCCGACGATTTGTTT	84	[36]
	R: TAGCGTCATCATTGGCTGGGA		

Relative gene expression was quantified by real-time PCR (RT-qPCR) using the ABI 7500 Fast Real-Time PCR System (Applied Biosystems). All reactions were performed in technical triplicates for each biological replicate using the SYBR^®^ Select Master Mix. Each 10 µL reaction contained 1 µL of cDNA (100 ng µL⁻¹), 0.3 µL of each primer (3 µM), 5 µL of SYBR^®^ Green Master Mix, and 3.4 µL of nuclease-free water.

Thermal cycling conditions consisted of an initial incubation at 50 °C for 2 min, followed by 40 amplification cycles at 95 °C for 15 s and 60 °C for 30 s. Fluorescence signals were acquired at the end of each extension step. A melting curve analysis (72–95 °C) was performed after amplification to verify product specificity, confirmed by the presence of a single dissociation peak. Amplification efficiency was determined using standard curves generated from serial cDNA dilutions (1:5) with acceptable efficiencies ranging from 90% to 110%. The RPS2 gene was used as the endogenous reference gene for normalization of RT-qPCR data. The 25 S rRNA and RPS2 genes were evaluated for melting curve analysis and amplification efficiency, as previously validated for Musa spp [35, 36].

### Statistical analysis

Relative expression data were subjected to analysis of variance (ANOVA) to assess treatment effects. Differences were considered statistically significant at *p* < 0.05. All analyses were performed using R software (version 4.5.2).

## Results

### Histological and histochemical responses

**Fig. 1:**
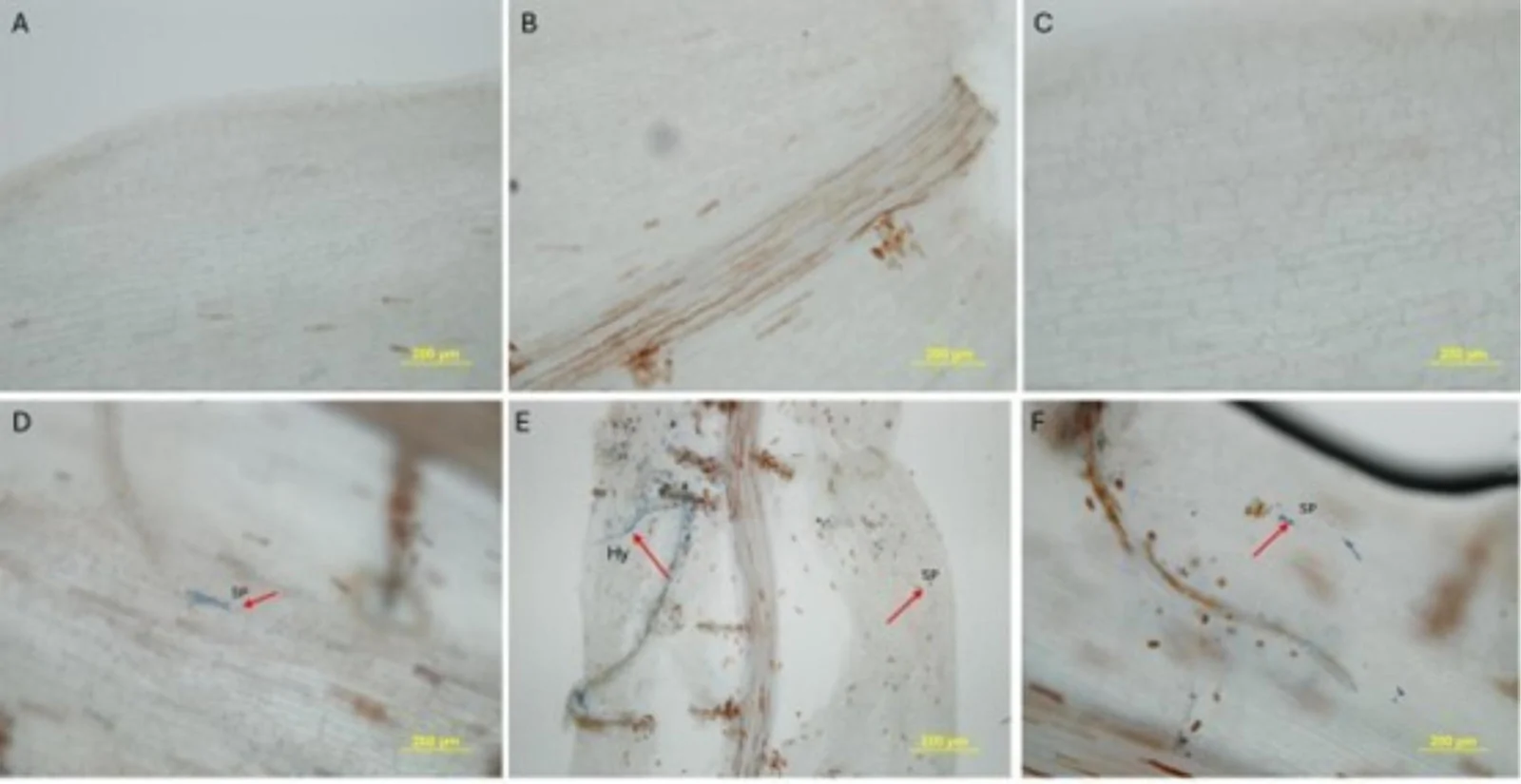
Analysis of *Foc* colonization in roots of the ‘Prata Ana’ genotype subjected to the bleaching and Trypan blue staining technique. Control (**A**) 24 HAI (uninoculated); Control (**B**) 48 HAI (uninoculated); Control (**C**) 72 HAI (uninoculated); (**D**) 24 HAI (inoculated); (**E**) 48 HAI (inoculated); (**F**) 72 HAI (inoculated). Hy: hyphae; SP: spores. Images were captured using a 10x objective lens. Bars = 200 μm

To verify the penetration of the fungus and its structures into the root tissues of the ‘Prata Ana’ genotype, the roots were subjected to a histopathological study using bleaching and Trypan blue staining techniques. The colonization of Foc, isolate CNPMF 218, in the root tissues of ‘Prata Ana’ is shown in Fig. 1 (A–F).

**Fig. 2:**
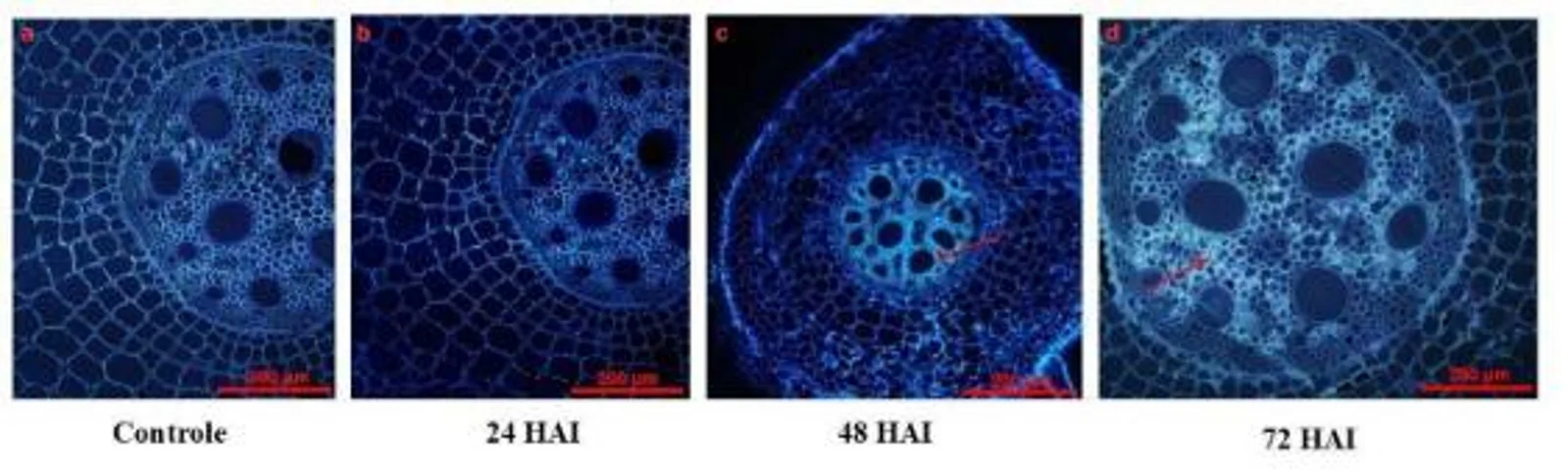
**(A–D**): Histochemical analysis by fluorescence microscopy (aniline blue staining) of cross-sections of ‘Prata-Ana’ banana roots showing the structural defense response. (**A**) Control treatment (uninoculated); **B**, **C**, and **D**: Samples inoculated with *Foc* collected at 24, 48, and 72 h after inoculation (HAI), respectively. The red arrows indicate points of fluorescence corresponding to callose deposition in the cell walls of the cortical parenchyma and xylem vessels. Images were captured using a 10x objective lens. Bars = 200 μm

The structural defense response was assessed through callose deposition. Callose is an important polysaccharide in reinforcing the cell wall and blocking fungal penetration, especially in phloem vessels [37, 38] Fig. 2 (A–D) illustrates the intensity and location of these physical barriers formed over time.

**Fig. 3:**
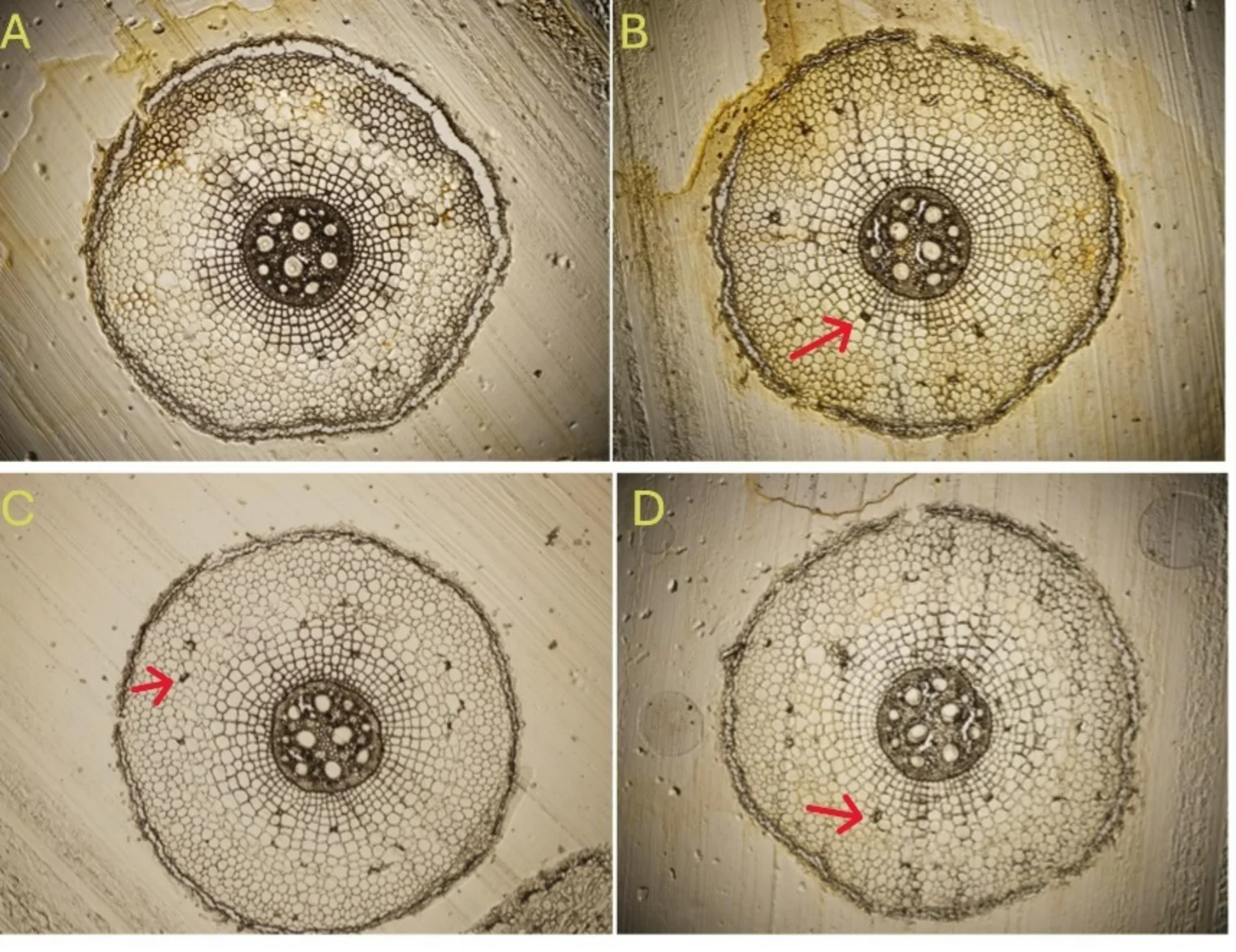
**A–D**: Histochemical test using ferric chloride to detect phenolic compounds in cross-sections of banana roots (Musa spp.) following inoculation with *Fusarium oxysporum* f. sp. *Cubense* (isolate CNPMF 218 - subtropical race 4). (**A**) Uninoculated control; (**B**) 24 h after inoculation (HAI); (**C**) 48 HAI; and (**D**) 72 HAI. The red arrows indicate darkly stained areas associated with the concentration of phenolic compounds in the cells of the cortical parenchyma

Histochemical analysis with ferric chloride revealed the presence of phenolic compounds identified as dark brown to black spots or deposits in the root tissue (Fig. 3: A–D).

### Foc symptoms

**Fig. 4:**
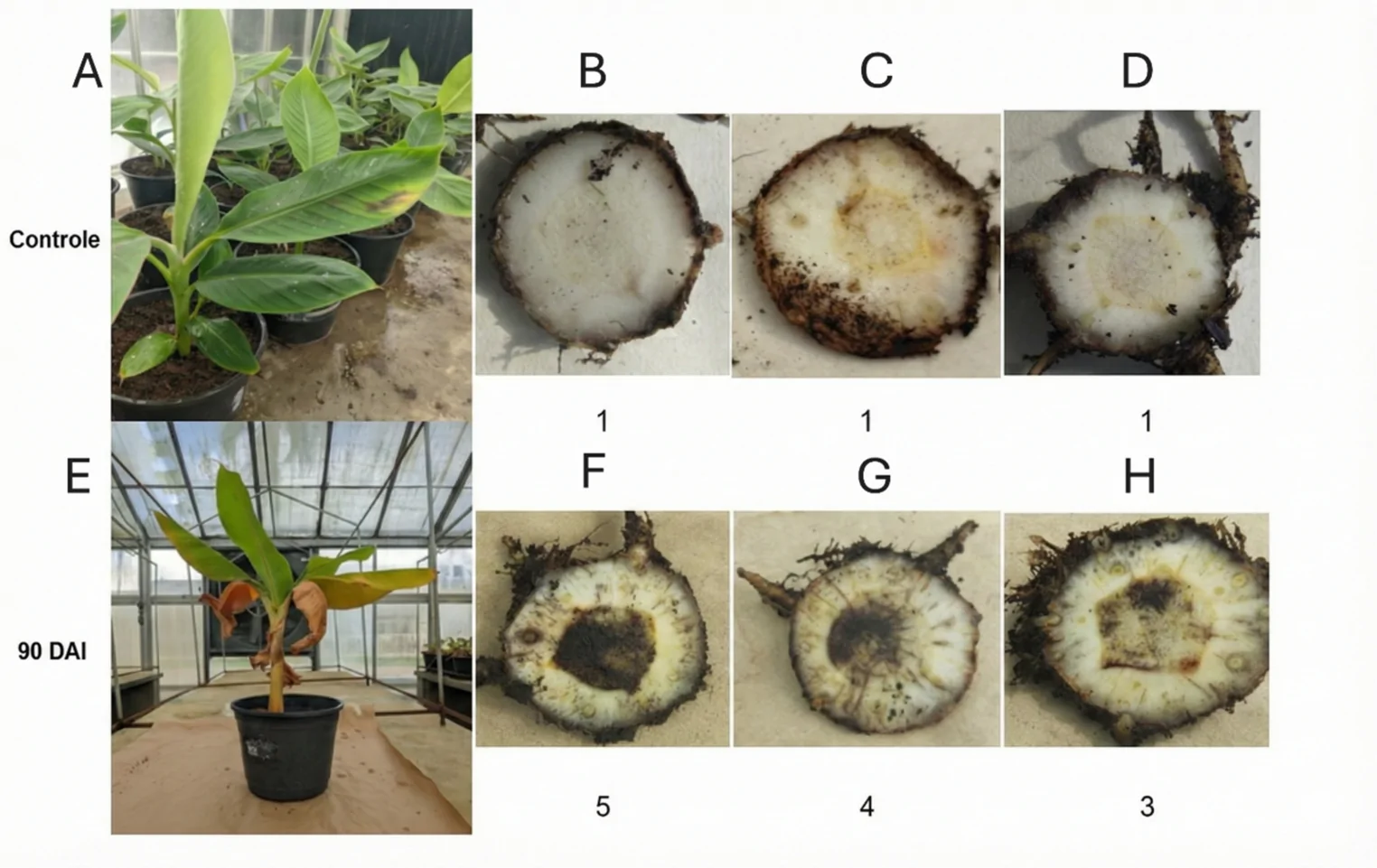
**(A–H).** Assessment of the severity of external and internal symptoms of Fusarium wilt in ‘Prata-Ana’ banana plants 90 days after inoculation (DAI). (**A–D**) Control treatment (uninoculated): **A**) General appearance of a healthy plant; **B**), **C)**, and **D**): Cross-sections of the rhizome showing infection-free tissues (score 1); (**E–H**): Plants inoculated with *Foc*; **E**) Plant exhibiting typical external symptoms of wilt and chlorosis; **(F, G, **and **H**): Cross-sections of the rhizome showing different levels of vascular necrosis progression, classified as grades 5, 4, and 3, respectively

The evaluation conducted 90 days after inoculation (DAI) confirmed the severity of the infection in the evaluated cultivar. Inoculated plants exhibited the classic symptoms of *Foc*, characterized by leaf chlorosis, necrosis, and wilting (Fig. 4: A–H). Internal analysis, performed using cross-sections of the rhizome revealed vascular discoloration ranging from dark brown to black, concentrated in the central cylinder and progressing toward the cortex, indicating necrosis of the vascular bundles and systemic colonization by the pathogen. Disease severity was quantified by assigning scores from 1 to 5 for the internal discoloration of the biological replicates, resulting in an average score of 4.0.

The Internal Damage Index (IDI) calculated for the genotype was 80%, which characterizes a highly severe compatible interaction. In contrast, the plants in the control treatment remained completely asymptomatic, exhibiting healthy rhizomes with a score of 1.0 (IDI = 0%), demonstrating the efficacy of the inoculation method and the integrity of the experiment. (Fig. 4: A–H).

### Validation of primers and amplification parameters

The integrity of the biological samples and the efficiency of cDNA synthesis were initially verified by amplification of the endogenous reference genes *25 S* and *RPS2*. Melting curve analysis for both genes showed a single well defined peak indicating specific amplification and the absence of inhibitors. For the target gene *MusaDMR6*, a single distinct melting peak was also observed confirming primer specificity. Amplification efficiencies were 118.6% for the reference gene *25 S* and 121.1% for *MusaDMR6*. Despite being slightly above the optimal range, efficiencies were comparable between genes, supporting the use of the 2^−ΔΔCt method for relative expression analysis [39–42].

### Gene expression analysis

Quantification of MusaDMR6 gene transcript levels was performed by real-time PCR (RT-qPCR) using the 2^-ΔΔCt comparative method. For each time point analyzed, relative expression was calculated by comparing samples inoculated with Foc to their respective non-inoculated controls, using the RPS2 gene as an endogenous control.

Relative expression was determined using the following equations:

$$\:{\Delta\:}Ct=C{t}_{\mathrm{MusaDMR6}}-C{t}_{\mathrm{RPS2}}$$ $$\:{\Delta\:}{\Delta\:}Ct={\Delta\:}C{t}_{\mathrm{amostra}}-{\Delta\:}C{t}_{\mathrm{controle\:basal\:(0h)}}$$ $$\:RQ={2}^{-{\Delta\:}{\Delta\:}Ct}$$

The mean cycle threshold (Ct) values for the MusaDMR6 gene in each treatment are presented in Table 2.

**Table 2:** Mean cycle threshold (Ct) values for the *MusaDMR6* gene in ‘Prata-Ana’ banana roots subjected to inoculation with *Foc* and control treatment (water)

Time (hpi)	Condition	Ct Mean (MusaDMR)
Time 0	Control	26.254
24 h	Inoculated (Foc)	26.40
24 h	Control (Water)	29.37
48 h	Inoculated (Foc)	32.46
48 h	Controle (Water)	30.96
72 h	Inoculated (Foc)	28.33
72 h	Control (Water)	30.92

**Fig. 5:**
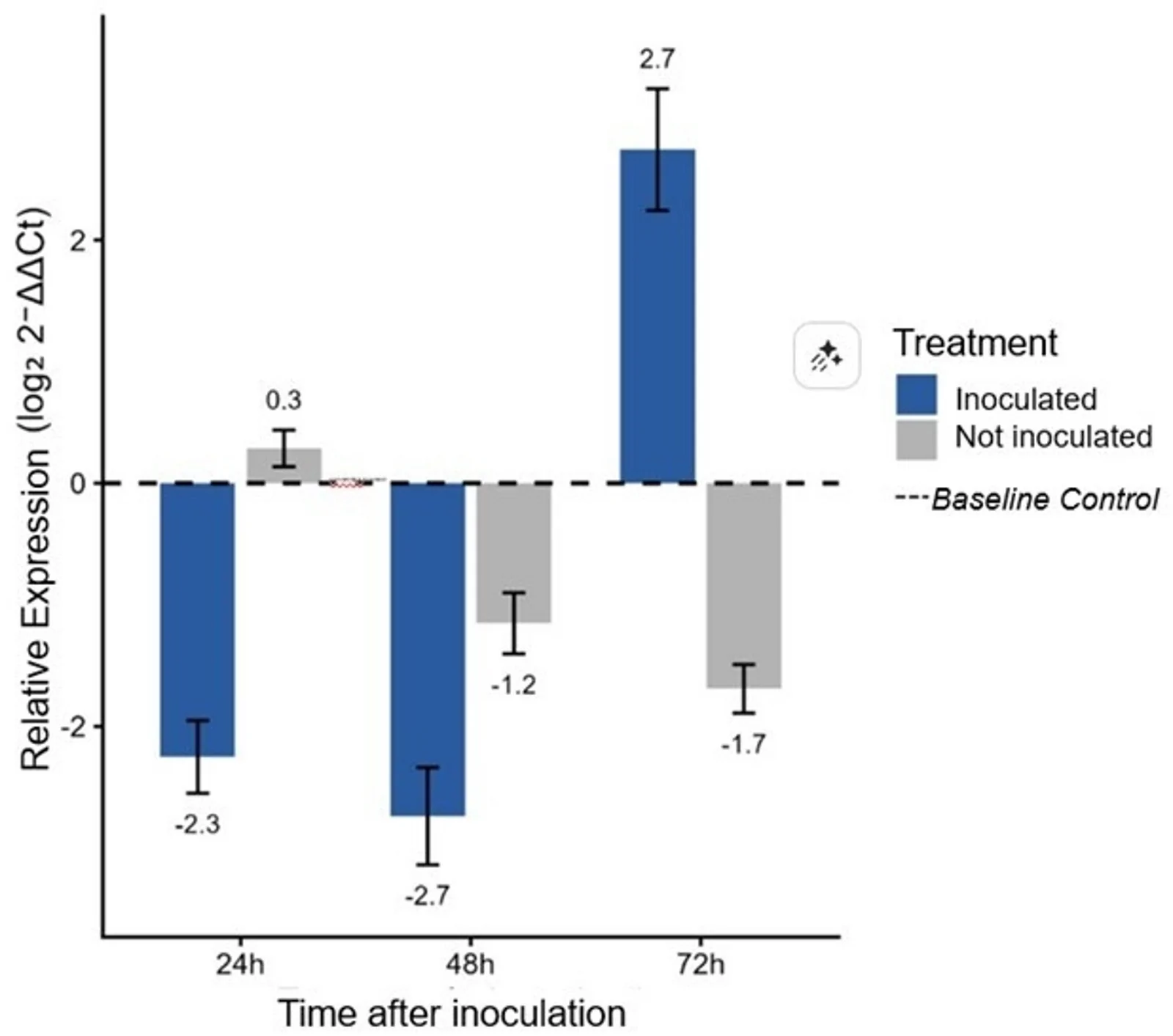
Relative expression profile (log₂(RQ)) of the *MusaDMR6* gene in ‘Prata-Ana’ banana roots inoculated with *Fusarium oxysporum* f. sp. *cubense* (Foc) and in non-inoculated controls (water)

To facilitate visualization of expression variations, RQ values were transformed to a base-2 logarithmic scale (Fig. 5), according to the following equation:

$$\:{\mathrm{l}\mathrm{o}\mathrm{g}}_{2}\left(RQ\right)=-{\Delta\:}{\Delta\:}Ct$$

### Expression profile of *MusaDMR6* during infection

A significant downregulation of *MusaDMR6* was observed during the early stages of infection with log₂(RQ) values of − 2.3 at 24 h and − 2.7 at 48 h post-inoculation in infected plants. In contrast, a marked upregulation was detected at 72 h, with a log₂(RQ) value of 2.7, corresponding to an approximately 6.5-fold increase in expression relative to the baseline control.

In non-inoculated plants, *MusaDMR6* expression remained near baseline levels or showed slight repression of the evaluated time points indicating that the observed transcriptional modulation is specifically associated with pathogen interaction. Mean cycle threshold (Ct) values for *MusaDMR6* ranged from 26.40 to 32.46 in inoculated samples and from 29.37 to 30.96 in control samples (Table 2).

### Analysis of Variance (ANOVA) of *MusaDMR6* Expression

**Fig. 6:**
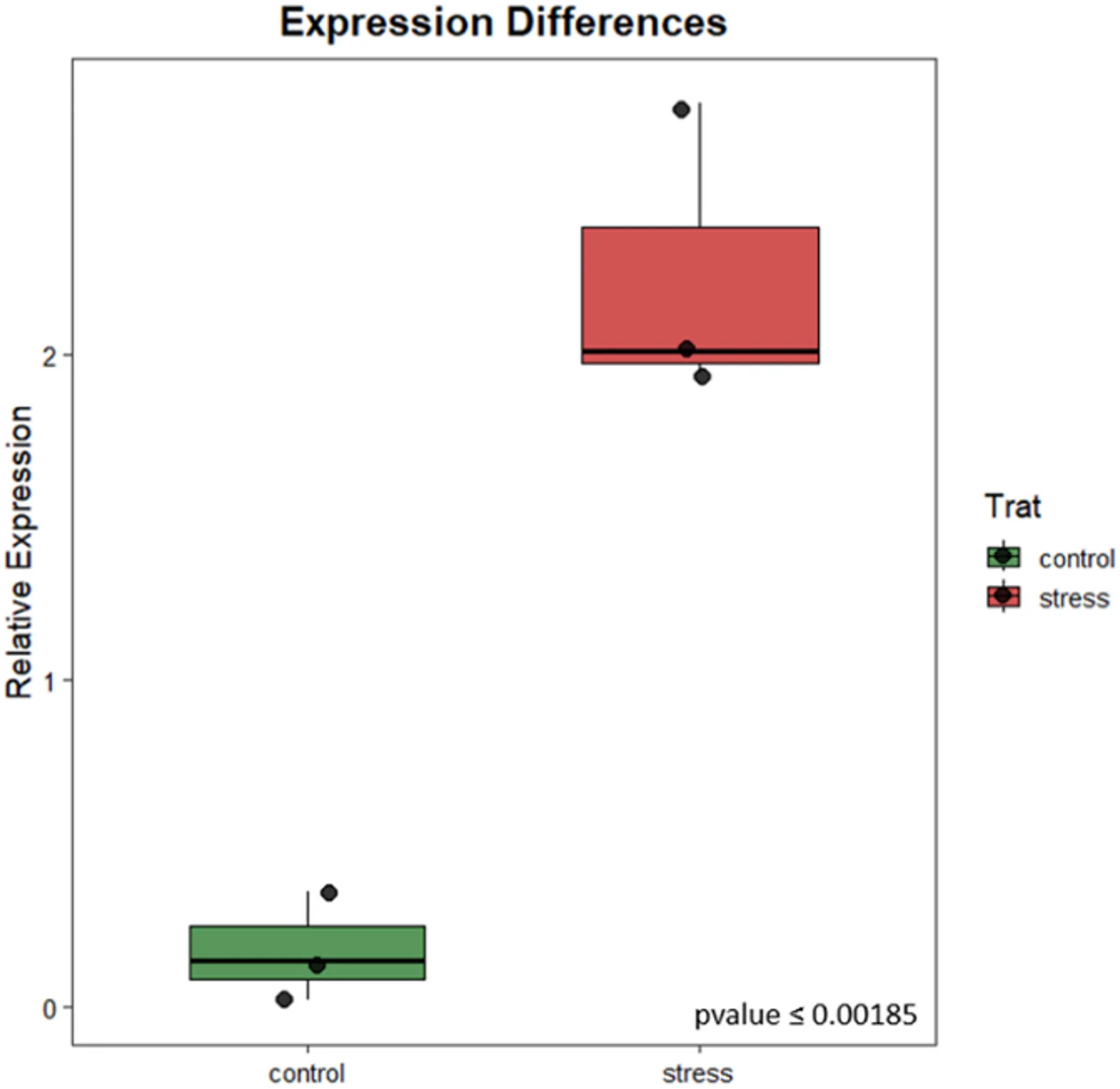
Analysis of variance (ANOVA) and boxplot of *MusaDMR6* relative expression in banana (Prata Ana) roots 72 h after inoculation with Foc

Analysis of variance (ANOVA) confirmed statistically significant differences between treatments (control and inoculated) at 72 h after inoculation (HAI) (Fig. 6). The test yielded a value of *p* = 0.00185, indicating high statistical significance (*p* < 0.01) for the observed gene induction.

## Discussion

Conventional strategies for genetic improvement of banana (*Musa* spp.) against Fusarium wilt have largely relied on the identification and introgression of dominant resistance (R) genes [3]. Although effective in some contexts, this approach is constrained by the biological characteristics of banana, including sterility, polyploidy, and long generation cycles, which limit breeding efficiency and frequently compromise fruit quality [9, 43]. Moreover, resistance based on single R-genes is inherently unstable, as demonstrated by the emergence of highly virulent races such as Tropical Race 4 (TR4) capable of overcoming previously resistant cultivars [44].

In this context, targeting susceptibility genes (S-genes) represents a complementary and potentially more durable strategy. Rather than enhancing recognition, this approach disrupts host factors required for pathogen compatibility [45, 46]. The present study focused on MusaDMR6, a gene encoding a 2-oxoglutarate-dependent oxygenase involved in the negative regulation of salicylic acid (SA)-mediated defense [20]. Its induction during pathogen challenge has been associated with suppression of basal immunity and facilitation of infection in multiple plant species [20, 21, 24–26].

The transcriptional profile observed here revealed an initial repression of MusaDMR6 at 24–48 h post-inoculation, followed by strong induction at 72 h. This biphasic pattern likely reflects the dynamic balance between host defense activation and pathogen-mediated immune suppression. Early repression may represent an attempt to maintain SA accumulation and restrict fungal establishment. However, the subsequent overexpression suggests that Fusarium oxysporum f. sp. cubense (Foc) successfully reprograms host transcriptional machinery, promoting DMR6-mediated SA catabolism and weakening defense responses. This transition coincides with the progression from early colonization to systemic infection, consistent with the hemibiotrophic lifestyle of Foc.

Histochemical analyses were performed to characterize the progression of the Musa–Foc interaction and confirmed successful pathogen colonization through the accumulation of phenolic compounds, callose deposition, and vascular discoloration [47]. These responses indicate that pathogen recognition and defense activation occurred during infection.

However, the distinction between resistance and susceptibility depends largely on the timing and magnitude of these responses rather than on their occurrence alone [4, 5]. Virulent Foc isolates can overcome these defense responses, allowing successful colonization of susceptible host tissues [3, 5]. Consequently, although these defense responses were activated, they were insufficient to prevent fungal colonization in the susceptible cultivar ‘Prata-Ana’.

Within this biological context, the transcriptional profile of *MusaDMR6* is consistent with its proposed role as a negative regulator of salicylic acid (SA)-mediated immunity [20, 21]. The marked induction of *MusaDMR6* at 72 h post-inoculation suggests that this gene may contribute to attenuating SA-mediated defense responses during pathogen establishment.

The extensive vascular discoloration and symptom severity observed at later stages further confirm successful pathogen establishment. The oxidation of phenolic compounds and lignification processes, although indicative of defense activation, ultimately contribute to xylem blockage and wilting symptoms. This reflects a typical compatible interaction, in which host responses are activated but fail to contain pathogen spread.

From a molecular perspective, the consistent induction of MusaDMR6 in inoculated plants, coupled with lower Ct values relative to controls, reinforces its role as a susceptibility factor actively exploited during infection. Although amplification efficiencies exceeded the ideal range, their similarity between target and reference genes supports the validity of relative quantification using the 2^−ΔΔCt method [48]. Slight efficiency inflation is likely attributable to inhibitory compounds commonly present in banana root tissues, such as polyphenols and polysaccharides [49].

The functional relevance of DMR6 is conserved across species. In bananas, its knockout has already conferred resistance to bacterial pathogens such as *Xanthomonas campestris pv. musacearum* and *Ralstonia syzygii* [21, 26]. Similar results in other crops, including potato and tomato, demonstrate that disruption of DMR6 enhances resistance to hemibiotrophic pathogens without major fitness penalties [24, 25]. Our findings extend these observations to the *Musa*–Foc interaction, suggesting that increased *MusaDMR6* expression is associated with susceptibility to Fusarium wilt. Importantly, the manipulation of S-genes such as DMR6 offers the potential for broad-spectrum resistance [25]. Additional targets, including members of the SWEET transporter family, WRKY transcription factors, and genes involved in ethylene signaling and translation initiation (e.g., eIF4E), have been implicated in susceptibility pathways and represent promising candidates for combinatorial editing strategies [50].

Because conventional breeding in commercial bananas is severely hindered by polyploidy, parthenocarpy, and high sterility, targeted gene editing has emerged as the most efficient approach for introducing resistance traits [7, 9]. Given the economic importance of ‘Prata-Ana’ in Brazil and its susceptibility to Fusarium wilt [43], the identification of MusaDMR6 as a transcriptionally responsive susceptibility gene provides a strong foundation for precision breeding. CRISPR/Cas9 mediated knockout of this gene represents a viable strategy to restore SA homeostasis and enhance basal immunity without altering desirable agronomic traits [14, 26].

Recent advances have already demonstrated that CRISPR/Cas9 can be successfully applied to Musa cultivars to confer robust disease resistance. Furthermore, this strategy preserves their original characteristics and fruit quality, which is crucial for consumer acceptance and market viability [13, 26]. It should be noted that this study provides transcriptional evidence of gene involvement but does not constitute functional validation. Future work should focus on generating knockout lines and evaluating resistance under greenhouse and field conditions, as well as assessing potential pleiotropic effects. Because the complete loss of function of immunity associated S genes can sometimes trigger a trade-off between defense and growth, potentially leading to stunted phenotypes or yield losses, it is crucial to monitor edited lines throughout their entire developmental cycle [45].

It is important to highlight that, because CRISPR/Cas9-mediated knockouts can generate transgene-free plants, these edited varieties currently benefit from increasingly favorable regulatory frameworks in several countries, including the guidelines established by the Comissão Técnica Nacional de Biossegurança (CTNBio) in Brazil. This permissive regulatory landscape, combined with the high precision of S gene editing, significantly reduces the time and cost from laboratory bench to commercial release. Thus, it provides an accelerated and realistic pathway to protect the banana industry against the threat of Fusarium wilt [13].

Furthermore, the expression dynamics of MusaDMR6 observed during Foc infection support its role as a key susceptibility factor in ‘Prata-Ana’. These findings reinforce the relevance of S-gene targeting as a strategic shift in banana breeding and provide a molecular basis for the development of durable resistance to Fusarium wilt through genome editing.

## Conclusions and future perspectives

This study identified *MusaDMR6* as a promising susceptibility gene associated with the interaction between ‘Prata-Ana’ and *Fusarium oxysporum* f. sp. *cubense*. Together with the histochemical evidence confirming successful pathogen colonization, the transcriptional profile observed here provides a molecular basis for genome editing strategies aimed at improving Fusarium wilt resistance in banana, identifying *MusaDMR6* as a promising target for precision breeding [3, 20, 21, 27].

Future studies should focus on the functional validation of *MusaDMR6* through CRISPR/Cas9-mediated knockout, followed by the evaluation of edited plants under greenhouse and field conditions. In addition, the identification and validation of other susceptibility genes, including members of the MLO, PMR4, SWEET, and WRKY families, may expand the repertoire of targets available for developing durable resistance to Fusarium wilt in banana [21, 22, 45]. Integrating functional genomics with genome editing will contribute to accelerating the development of resistant cultivars while preserving the agronomic characteristics of commercially important banana varieties.

## Data Availability

No datasets were generated or analysed during the current study.
